# Cost-Effectiveness of Poly ADP-Ribose Polymerase Inhibitors in Cancer Treatment: A Systematic Review

**DOI:** 10.3389/fphar.2022.891149

**Published:** 2022-07-11

**Authors:** Vivien Kin Yi Chan, Runqing Yang, Ian Chi Kei Wong, Xue Li

**Affiliations:** ^1^ Department of Pharmacology and Pharmacy, Li Ka Shing Faculty of Medicine, University of Hong Kong, Hong Kong SAR, China; ^2^ Department of Medicine, School of Clinical Medicine, Li Ka Shing Faculty of Medicine, University of Hong Kong, Hong Kong SAR, China; ^3^ Research Department of Policy and Practice, School of Pharmacy, University College London, London, United Kingdom; ^4^ Laboratory of Data Discovery for Health (D^2^4H), Hong Kong Science Park, Hong Kong SAR, China; ^5^ HKU-Shenzhen Hospital, Shenzhen, China

**Keywords:** cost-effectiveness, systematic review, PARP inhibitors, precision oncology, health economics, health policy

## Abstract

**Background:** PARP inhibitors have shown significant improvement in progression-free survival, but their costs cast a considerable financial burden. In line with value-based oncology, it is important to evaluate whether drug prices justify the outcomes.

**Objectives:** The aim of the study was to systematically evaluate PARP inhibitors on 1) cost-effectiveness against the standard care, 2) impact on cost-effectiveness upon stratification for genetic characteristics, and 3) identify factors determining their cost-effectiveness, in four cancer types.

**Methods:** We systematically searched PubMed, EMBASE, Web of Science, and Cochrane Library using designated search terms, updated to 31 August 2021. Trial-based or modeling cost-effectiveness analyses of four FDA-approved PARP inhibitors were eligible. Other studies known to authors were included. Reference lists of selected articles were screened. Eligible studies were assessed for methodological and reporting quality before review.

**Results**: A total of 20 original articles proceeded to final review. PARP inhibitors were not cost-effective as recurrence maintenance in advanced ovarian cancer despite improved performance upon genetic stratification. Cost-effectiveness was achieved when moved to upfront maintenance in a new diagnosis setting. Limited evidence indicated non–cost-effectiveness in metastatic breast cancer, mixed conclusions in metastatic pancreatic cancer, and cost-effectiveness in metastatic prostate cancer. Stratification by genetic testing displayed an effect on cost-effectiveness, given the plummeting ICER values when compared to the “treat-all” strategy. Drug cost was a strong determinant for cost-effectiveness in most models.

**Conclusions**: In advanced ovarian cancer, drug use should be prioritized for upfront maintenance and for patients with BRCA mutation or BRCAness at recurrence. Additional economic evaluations are anticipated for novel indications.

## 1 Introduction

The development of poly (ADP-ribose) polymerase (PARP) inhibitors represents a breakthrough in first harnessing the “synthetic lethality” concept in clinical use (Helleday, 2011; Sonnenblick et al., 2015) and kick-started the era of redefining a single tumor type for stratification into distinct diseases specific to genetic aberrations. Patients with tumor-harboring BRCA1/2 mutations or who show homologous recombination deficiency (HRD) are particularly sensitive to the effect of PARP inhibitors (O'Sullivan et al., 2014). At the time of writing, four PARP inhibitors have been approved by the U.S. Food and Drug Administration (FDA): olaparib, niraparib, rucaparib, and talazoparib (RUBRACA (rucaparib), 2020; LYNPARZA (olaparib), 2021; ZEJULA (niraparib) 2021; TELZENNA (talaparib), 2021).

Although efficacy as first-line monotherapy is as yet unproven, PARP inhibitors as maintenance therapy amplify the existing treatment effect. In advanced ovarian cancer, patients receive repeated courses of platinum-based chemotherapies with over 70% risk of recurrence until “platinum resistance” (Jiang et al., 2019; Ovarian Cancer Research Alliance, 2020). In metastatic pancreatic cancer, progression-free survival (PFS) following first-line chemotherapies last only 6 months with less than 10% of patients surviving after 5 years (Conroy et al., 2018; Rawla et al., 2019). PFS often diminishes with subsequent cycles; maintenance therapy between lines could prolong PFS and allow patient eligibility for subsequent strategies, thus enhancing survival likelihood (Evans and Matulonis, 2017). PARP inhibitors targeting ovarian cancer all demonstrated longer median PFS against placebo (olaparib, niraparib, and rucaparib: 16.6–21.0 vs. 5.4–5.5 months) in BRCAmut cohorts of recurrent platinum-sensitive cases, and in the first-line maintenance setting, olaparib and niraparib further extended PFS by 3 years and 1 year among BRCAmut and HRD-positive patients, respectively (Ledermann et al., 2012; Mirza et al., 2016; Coleman et al., 2017; Pujade-Lauraine et al., 2017; Moore et al., 2018; González-Martín et al., 2019). Patients with gBRCAmut metastatic pancreatic cancer also had longer PFS with maintenance olaparib against placebo (7.4 vs. 3.8 months) (Golan et al., 2019). Apart from maintenance, PARP inhibitors demonstrated efficacy in later lines as active treatment for gBRCAmut metastatic breast cancer (PFS extension with olaparib and talazoparib: 2.8–3 months) and gBRCAmut and/or HRD-positive metastatic castration-resistant prostate cancer (PFS with olaparib vs. placebo: 7.4 vs. 3.6 months; objective response rate with rucaparib: 43.5–50.8%) (Robson et al., 2017; Litton et al., 2018; Abida et al., 2020; Hussain et al., 2020).

Value-based oncology is thus warranted to address the cost-effectiveness of novel drugs, for which acceptable prices should be tied to justifiable patient outcomes by cost-effectiveness analyses (Neumann et al., 2021). Incremental cost-effectiveness ratio (ICER), a quotient of the cost difference between two therapeutic interventions divided by the outcome difference, denotes the incremental monetary value for an additional life-year or quality-adjusted life-year (QALY). When this falls below the willingness-to-pay (WTP) or when a strategy is both cost-saving and clinically superior (dominance), it is concluded as cost-effective. A previous literature review on the cost-effectiveness studies of PARP inhibitors focused, however, only on methodological quality and publications related to ovarian cancer (Gao et al., 2020).

Given the recently approved multiple indications in a variety of cancer types and the inconsistent genetic prerequisites for BRCA mutation and HRD status across indications, it is also questionable whether the full biomarker-guided use of PARP inhibitors would improve cost-effectiveness as they acted more profoundly on patient stratification. In this systematic review, we aimed to evaluate PARP inhibitors on 1) the cost-effectiveness against the standard of care, 2) impact on cost-effectiveness upon stratification for genetic characteristics, and 3) to elucidate the key factors that determine cost-effectiveness in the management of ovarian, breast, pancreatic, and prostate cancers.

## 2 Methods and Materials

This study was conducted according to the recommended checklist of the Preferred Reporting Items for Systematic Reviews and Meta-Analyses (PRISMA) guidelines and benchmarked with the methodology of similar systematic reviews on medicinal cost-effectiveness (Moher et al., 2009; Verma et al., 2018; Yoshida et al., 2020).

### 2.1 Search Strategy and Eligibility

We systematically searched PubMed, EMBASE, Web of Science, and Cochrane Library, without language and date restriction, using the search terms: (“poly ADP-ribose polymerase inhibitors” OR “PARP inhibitors” OR “olaparib” OR “rucaparib” OR “niraparib” OR “talazoparib”) and (“cost” OR “cost-effectiveness” OR “cost-utility” OR “economics”) in any field, updated to 31 August 2021. Reference lists of eligible articles were checked for additional relevant articles, and other studies known to the authors were included. Eligibility criteria were trial-based, or modeling cost-effectiveness analyses published in English or Chinese language related to any of the four FDA-approved PARP inhibitors, regardless of cancer types, lines of treatment, and comparator interventions. Non-comparative studies, reviews, responses, editorials, protocols, and abstract-only articles were excluded.

### 2.2 Quality Assessment

Studies were assessed using the Quality of Health Economics Studies (QHES) instrument (for methodological quality) and the Consolidated Health Economic Evaluation Reporting Standards (CHEERS) checklist (for reporting quality) (Ofman et al., 2003; Husereau et al., 2013). Articles which obtained a QHES score above 74 out of 100 and CHEERS score above 20 out of 24 were qualified for final data extraction and synthesis.

### 2.3 Data Extraction and Synthesis

Data were extracted based on a pre-defined extraction framework for bibliography, methods, results, and conclusion, including ICER at base-case analysis and model impact at sensitivity analyses. All presented monetary values were converted to U.S. dollars in the year of publication. The primary outcome of interest was the ICER of PARP inhibitors compared with observation (no maintenance treatment after standard treatment), alternative PARP inhibitors, and the standard of care.

Two independent researchers (VKYC and RQY) performed literature screening and quality assessment. Data were extracted by one researcher (RQY) and cross-checked by another researcher (VKYC). All discrepancies were resolved in consensus meetings.

## 3 Results

### 3.1 Study Selection and Quality Assessment

**FIGURE 1:**
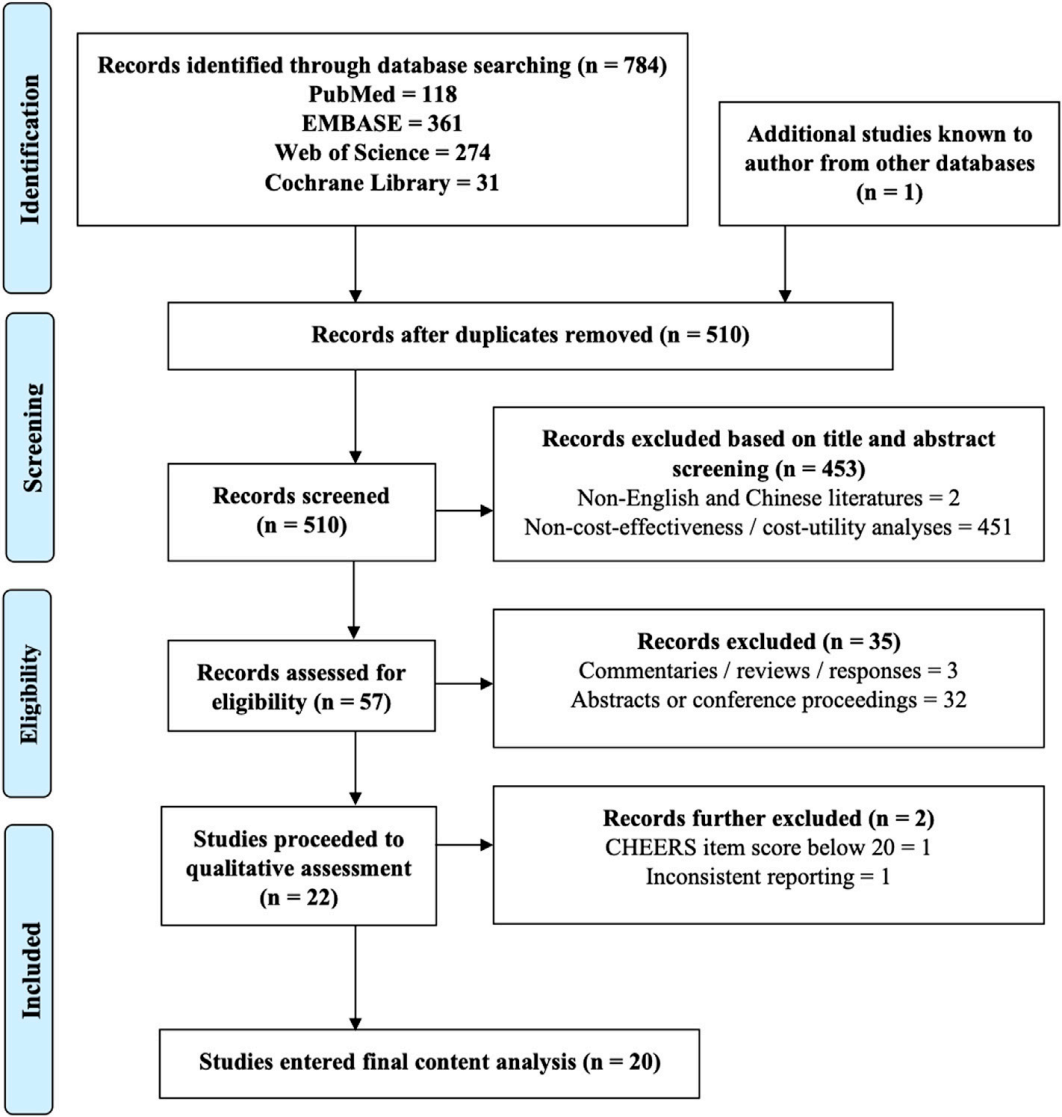
Literature screening process based on 2018 PRISMA flow diagram.

A total of 22 original full-text studies passed the initial screening for eligibility (Figure 1). Among them, 21 articles achieved good methodological and reporting quality (mean QHES score: 92.5/100 and a CHEERS score of 22.5/24) (Supplementary Table S1). One study was excluded further due to inconsistent reporting. Eventually, 20 articles proceeded into final review.

### 3.2 Study Characteristics

**FIGURE 2:**
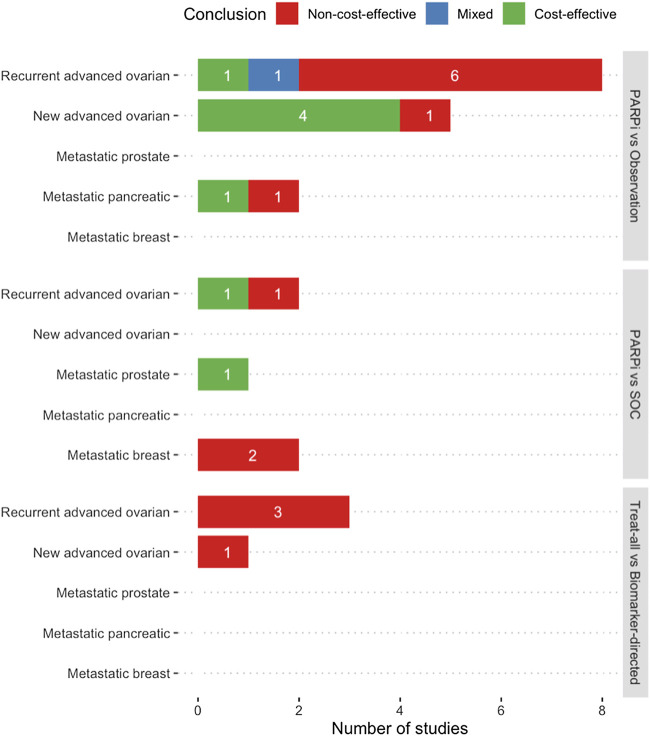
Summary of economic evaluation outcomes of included studies (a,b). (a) Mixed conclusion indicates the presence of both positive and negative conclusion for cost-effectiveness in different comparison arms in the same study. (b) PARPi vs Observation = Any PARP inhibitors were compared against observation, routine surveillance, or placebo, which all mean no maintenance therapy after standard chemotherapy. PARPi vs SOC = Any PARP inhibitors were compared against standard chemotherapy or hormone therapy. Treat-all vs Biomarker-directed = An approach when any PARP inhibitors were given to all patients without genetic characterization, compared with treatment on selective patients guided by the results of genetic tests. Abbreviations: PARPi − PARP inhibitors, SOC − Standard of care.

Table 1 illustrates the general characteristics of the included studies. The majority of studies were conducted in the United States (*n* = 13), five in Asia, and two in Europe. Most studies targeted patients with advanced ovarian cancer (*n* = 15), with nine focusing on recurrence and six covering new diagnosis setting. The remaining studied metastatic pancreatic (*n* = 2), breast (*n* = 2), and prostate (*n* = 1) cancers. Five studies investigated the role of PARP inhibitors as active treatment, 16 studies as maintenance treatment, and one covered both categories. All studies used decision modeling. The most frequently adopted time horizon was a short-term between 1 and 5 years or until disease progression (*n* = 10). Ten studies were set out from the payer’s perspective, seven from a healthcare system perspective, and three from a societal perspective. Major comparison arms were observation (*n* = 15), followed by standard care (*n* = 5), and alternative biomarker-directed strategies (*n* = 2). All studies stratified patients based on BRCA mutation, but only three studies examined the effect of HRD status. Figure 2 summarizes the economic outcomes by comparison arms, and Tables 2– 4 provide detailed economic outcomes of each included study.

**TABLE 1:** Characteristics of included studies by indication.

Study	Year	Country and perspective	PARPi role	Comparison category^a^	Comparison arms	Model	Time horizon
Recurrent advanced ovarian cancer							
Secord et al.	2013	US society	Recurrence maintenance	PARPi vs. observation Treat-all’ vs. Biomarker-directed strategy	(1) *BRCA* testing followed by selective olaparib vs. observation (2) “Global olaparib” vs. *BRCA* testing followed by selective olaparib	Modified Markov model	12 months
Smith et al.	2015	US third-party payer	Recurrence maintenance	PARPi vs. observation	(1) Olaparib vs. observation (g*BRCA*mut) (2) Olaparib vs. observation (*BRCA*wt)	Decision analysis model	Not mentioned
Wallbillich et al.^b^	2016	US payer	Recurrence active treatment	PARPi vs. standard care	Genomic-guided targeted therapy^c^ vs. chemotherapy for all without testing	Decision model	12 months
Institute for Clinical and Economic Review Report	2017	US healthcare system	Recurrence maintenance and recurrence active treatment	PARPi vs. observation PARPi vs. standard care	(1) Olaparib vs. placebo (g*BRCA*mut) (2) Niraparib vs. placebo (g*BRCA*mut) (3) Niraparib vs. placebo (non-g*BRCA*mut) (4) Rucaparib vs. placebo (*BRCA*mut) (5) Olaparib vs. chemotherapy (g*BRCA*mut) (6) Rucaparib vs. chemotherapy (*BRCA*mut)	Semi-Markov model	15 years
Zhong et al.	2018	US healthcare sector	Recurrence maintenance	PARPi vs. observation PARPi vs. PARPi	(1) Olaparib vs. placebo (general population) (2) Niraparib vs. placebo (general population) (3) Olaparib vs. placebo (g*BRCA*mut) (4) Niraparib vs. placebo (g*BRCA*mut) (5) Olaparib vs. placebo (non-g*BRCA*mut) (6) Niraparib vs. placebo (non-g*BRCA*mut) (7) Niraparib vs. olaparib (general population) (8) Olaparib vs. niraparib (g*BRCA*mut) (9) Niraparib vs. olaparib (non-g*BRCA*mut)	Decision tree model	Until disease progression or death
Dottino et al.	2019	US society	Recurrence maintenance	PARPi vs. observation ‘Treat-all’ vs. biomarker-directed strategy	(1) g*BRCA* mutation testing followed by selective niraparib vs. observation (2) g*BRCA* mutation + tumor HRD testing followed by selective niraparib vs. g*BRCA* mutation testing followed by selective niraparib (3) Treat all with niraparib vs. g*BRCA* mutation + tumor HRD testing, followed by selective niraparib	Decision analysis model	Less than 24 months
Guy et al.	2019	US payer	Recurrence maintenance	PARPi vs. observation PARPi vs. PARPi	(1) Niraparib vs. routine surveillance (g*BRCA*mut)* (2) Niraparib vs. routine surveillance (non-g*BRCA*mut)* (3) Niraparib vs. olaparib (non-g*BRCA*mut) (4) Niraparib vs. rucaparib (g*BRCA*mut) (5) Niraparib vs. rucaparib (non-g*BRCA*mut)	Decision-analytic model	Lifetime
Cheng et al.	2021	SG healthcare system	Recurrence maintenance	PARPi vs. observation ‘Treat-all’ vs. biomarker-directed strategy	(1) Olaparib for all patients vs. observation (2) Olaparib for g*BRCA*mut patients only vs. observation (3) Olaparib for all patients vs. olaparib for g*BRCA*mut patients only	Partition-survival model	15 years
Leung et al.	2021	TW healthcare system	Recurrence maintenance	PARPi vs. observation	(1) Olaparib vs. placebo (all patients)* (2) Niraparib vs. placebo (all patients)* (3) Olaparib vs. placebo (g*BRCA*mut)* (4) Niraparib vs. placebo (g*BRCA*mut)* (5) Olaparib vs. placebo (non-g*BRCA*mut) (6) Niraparib vs. placebo (non-g*BRCA*mut)	Decision analysis model	24 months
Newly diagnosed advanced ovarian cancer							
Armeni et al.	2020	IT National health service	First-line maintenance	PARPi vs. observation	Olaparib vs. active surveillance (*BRCA*mut)*	Markov model	50 years (lifetime)
Barrington et al.	2020	US third-party payer	First-line maintenance	PARPi vs. observation	(1) Niraparib vs. observation (all patients)* (2) Niraparib vs. observation (HRD-positive)* (3) Niraparib vs. observation (*BRCA*mut)* (4) Niraparib vs. observation (HRD-positive, non-*BRCA*mut)* (5) Niraparib vs. observation (Non–HRD-positive)*	Decision analysis model	Not mentioned
Gonzalez et al.	2020	US third-party payer	First-line maintenance	‘Treat-all’ vs. biomarker-directed strategy	(1) Niraparib for all patients vs. biomarker-directed niraparib (2) Olaparib/bevacizumab for all patients vs. biomarker-directed olaparib/bevacizumab	Modified Markov model	28 months–45 months
Muston et al.	2020	US third-party payer	First-line maintenance	PARPi vs. observation	(1) Olaparib vs. surveillance (*BRCA*mut)*	Partition-survival model	50 years (lifetime)
Penn et al.	2020	US healthcare system	First-line maintenance	PARPi vs. observation	*BRCA*mut patients: (1) Olaparib vs. observation (2) Olaparib–bevacizumab vs. observation (3) Bevacizumab vs. observation (4) Niraparib vs. observation HRD-positive, non-*BRCA*mut patients: (5) Olaparib–bevacizumab vs. observation(6) Bevacizumab vs. observation (7) Niraparib vs. observation HRD-positive patients: (8) Olaparib–bevacizumab vs. observation (9) Bevacizumab vs. observation (10) Niraparib vs. observation	Decision tree model	2 years
Tan et al.	2021	SG healthcare payer	First-line maintenance	PARPi vs. observation	Olaparib vs. routine surveillance (*BRCA*mut)*	Partition-survival model	50 years (lifetime)
Germline *BRCA*-mutated, HER2-negative locally advanced or metastatic breast cancer							
Saito et al.	2019	JP payer	Recurrence active treatment	PARPi vs. standard care	Olaparib for *BRCA*mut patients after *BRCA* testing vs. standard chemotherapy alone	Markov cohort model	5 years
Lima et al.	2021	SP national health system	Recurrence active treatment	PARPi vs. standard care	After anthracyclines/taxanes: (1) Talazoparib vs. capecitabine After anthracyclines/taxanes and capecitabine: (2) Talazoparib vs. eribulin	Partition-survival model	43 months
Germline *BRCA*-mutated metastatic pancreatic cancer							
Wu et al.	2020	US payer	First-line maintenance	PARPi vs. observation	Olaparib vs. placebo*	Partition-survival model	Not mentioned
Zhan et al.	2020	CN society	First-line maintenance	PARPi vs. observation	Olaparib vs. placebo	Markov model	5 years
HRD-positive metastatic castration-resistant prostate cancer							
Su et al.	2021	US payer	Recurrence active treatment	PARPi vs. standard care	Patients with at least one alteration in *BRCA*1/2 and *ATM*: (1) Olaparib vs. standard care*Patients with alterations in any of 15 pre-specified genes: (2) Olaparib vs. standard care*	Partition-survival model	Not mentioned

Asterisk (*) indicates that a positive conclusion for cost-effectiveness was achieved. ^a^ *PARPi vs. Observation* = any PARP inhibitors were compared against observation, routine surveillance, or placebo, which all mean no maintenance therapy after standard chemotherapy. PARPi vs. SOC = any PARP inhibitors were compared against standard chemotherapy or hormone therapy. Treat-all vs. biomarker-directed = an approach when any PARP inhibitors were given to all patients without genetic characterization, compared with treatment on selective patients guided by the results of genetic tests. ^b^ Wallbillich et al. study was the only group that studied the use of PARPi among platinum-resistant patients. ^c^ PARPi acted as one of the consequential treatment choices guided by the genome-based diagnostic test. Abbreviations: HRD = homologous recombination deficiency; PARPi = poly (ADP-ribose) polymerase inhibitors; SG = Singapore; US = United States; JP = Japan; IT = Italy; CN = China; TW = Taiwan; SP = Spain.

**TABLE 2:** Details of economic evaluation outcomes of studies in recurrent advanced ovarian cancer.

Study	Comparison arms	IncreEff	IncreCost	Discount rate	ICER	WTP threshold	Conclusion
PARPi vs. observation							
Secord et al.	BRCA testing followed by selective olaparib vs. observation	0.7 months PFS^†^	$11,518^†^	None	$193,442 per PF-YLS	$100,000	Not CE
Smith et al.	Olaparib vs. observation (gBRCAmut)	6.9 months PFS	$147,477^†^	Not reported	$258,864 per PF-LYS	$50,000	Not CE
	Olaparib vs. observation (BRCAwt)	1.9 months PFS	$95,089^†^	Not reported	$600,552 per PF-LYS	$50,000	Not CE
Institute for Clinical and Economic Review Report	Olaparib vs. placebo (gBRCAmut)	0.59 QALY^†^	$192,114^†^	3% yearly	$324,116 per QALY	$50,000–150,000	Not CE
	Niraparib vs. placebo (gBRCAmut)	0.65 QALY^†^	$191,959^†^	3% yearly	$291,454 per QALY	$50,000–150,000	Not CE
	Niraparib vs. placebo (non-gBRCAmut)	0.07 QALY^†^	$126,966^†^	3% yearly	$1.9M per QALY	$50,000–150,000	Not CE
	Rucaparib vs. placebo (BRCAmut)	0.49 QALY^†^	$178,083^†^	3% yearly	$369,175 per QALY	$50,000–150,000	Not CE
Zhong et al.	Olaparib vs. placebo (general population)	0.43 PFS-LY	$122,000	None	$287,000 per PFS-LY	$100,000	Not CE
	Olaparib vs. placebo (gBRCAmut)	1.13 PFS-LYs^†^	$255,500^†^	None	$197,000 per PFS-LY	$100,000	Not CE
	Olaparib vs. placebo (non-gBRCAmut)	0.3 PFS-LY	$98,500	None	$328,000 per PFS-LY	$100,000	Not CE
	Niraparib vs. placebo (general population)	0.58 PFS-LY^†^	$136,800^†^	None	$235,000 per PFS-LY	$100,000	Not CE
	Niraparib vs. placebo (gBRCAmut)	1.29 PFS-LYs	$254,700	None	$226,000 per PFS-LY	$100,000	Not CE
	Niraparib vs. placebo (non-gBRCAmut)	0.46 PFS-LY	$116,000^†^	None	$253,000 per PFS-LY	$100,000	Not CE
Dottino et al.	gBRCA mutation testing followed by selective niraparib vs. observation	0.19 PF-QALY	$45,330	None	$243,092 per PF-QALY	$100,000	Not CE
Guy et al.	Niraparib vs. routine surveillance (gBRCAmut)	4.41 QALYs	$301,174	3% yearly	$68,287 per QALY	$150,000	CE
	Niraparib vs. routine surveillance (non-gBRCAmut)	2.148 QALYs	$232,598	3% yearly	$108,287 per QALY	$150,000	CE
Cheng et al.	Olaparib for all patients vs. observation	0.6627 QALY	$66,879	3% yearly	$100,926 per QALY	No fixed WTP	Not CE
	Olaparib for gBRCAmut patients only vs. observation	0.1637 QALY	$14,334	3% yearly	$87,566 per QALY	No fixed WTP	Not CE
Leung et al.	Olaparib vs. placebo (all patients)	0.46 PFS-LY	$29,805	3% yearly	$64,457 per PFS-LY	$92,943	CE
	Niraparib vs. placebo (all patients)	0.62 PFS-LY	$51,686	3% yearly	$83,581 per PFS-LY	$92,943	CE
	Olaparib vs. placebo (gBRCAmut)	1.13 PFS-LYs	$29,805	3% yearly	$26,329 per PFS-LY	$92,943	CE
	Niraparib vs. placebo (gBRCAmut)	1.29 PFS-LYs	$51,686	3% yearly	$40,005 per PFS-LY	$92,943	CE
	Olaparib vs. placebo (non-gBRCAmut)	0.29 PFS-LY	$29,805	3% yearly	$101,033 per PFS-LY	$92,943	Not CE
	Niraparib vs. placebo (non-gBRCAmut)	0.45 PFS-LY	$51,686	3% yearly	$114,859 per PFS-LY	$92,943	Not CE
PARPi vs. standard care							
Wallbillich et al.	Genomic test-guided targeted therapies vs. PLD for all without testing	0.03 QALY^†^	$15,345^†^	Not reported	$479,303 per QALY	$100,000	Not CE
Institute for Clinical and Economic Review Report	Olaparib vs. PLD + C (gBRCAmut)	0.67 QALY^†^	$96,864^†^	3% yearly	$146,210 per QALY	$50,000–150,000	CE
	Rucaparib vs. PLD + C (BRCAmut)	0.61 QALY^†^	$180,123^†^	3% yearly	$294,593 per QALY	$50,000–150,000	Not CE
“Treat-all” vs. biomarker-directed strategy							
Secord et al.	“Global olaparib” vs. BRCA testing, followed by selective olaparib	2.1 months PFS^†^	$39,822^†^	None	$234,128 per PF-YLS	$100,000	Not CE
Dottino et al.	gBRCA mutation + tumor HRD testing followed by selective niraparib vs. gBRCA mutation followed by selective niraparib	0.23 PF-QALY	$63,211	None	$269,883 per PF-QALY	$100,000	Not CE
	Treat all with niraparib vs. gBRCA mutation + tumor HRD testing, followed by selective niraparib	0.03 PF-QALY	$59,759	None	$2.2M per PF-QALY	$100,000	Not CE
Cheng et al.	Olaparib for all patients vs. olaparib for gBRCAmut only	0.499 QALY	$52,545	3% yearly	$105,308 per QALY	No fixed WTP	Not CE

All monetary values were converted into U.S. dollars, using specified exchange rates in publication or average exchange rates in the corresponding year of publication. CE = cost-effective; Not CE = not cost-effective. † Incremental values that were computed manually due to the lack of exact figures in original studies. Abbreviation: BRCAmut = BRCA mutation; BRCAwt = BRCA wild-type; gBRCAmut = germline BRCA mutation; HRD = homologous recombination deficiency; IncreCost = incremental cost; IncreEff = incremental effectiveness; LY = life-year; PARPi = poly(ADP-ribose) polymerase inhibitors; PLD(+C) = pegylated liposomal doxorubicin (+carboplatin); PF = progression-free; PFS = progression-free survival; QALY = quality-adjusted life year; WTP = willingness-to-pay.

**TABLE 3:** Details of economic evaluation outcomes of studies in newly diagnosed advanced ovarian cancer.

Study	Comparison arms	IncreEff	IncreCost	Discount rate	ICER	WTP threshold	Conclusion
PARPi vs. observation							
Armeni et al.	Olaparib vs. active surveillance (BRCAmut)	2.41 QALYs	$30,586	3% yearly	$12,703 per QALY; $10,654 per LY	$18,332	CE
Barrington et al.	Niraparib vs. observation (All patients)	5.6 months PFS	$918,750	Not mentioned	$72,829 per QALY	$100,000	CE
	Niraparib vs. observation (HRD-positive)	11.5 months PFS	$737,500	Not mentioned	$56,329 per QALY	$100,000	CE
	Niraparib vs. observation (BRCAmut)	11.2 months PFS	$412,500	Not mentioned	$58,348 per QALY	$100,000	CE
	Niraparib vs. observation (HRD-positive, non-BRCAmut)	11.4 months PFS	$300,000	Not mentioned	$50,914 per QALY	$100,000	CE
	Niraparib vs. observation (Non-HRD-positive)	2.7 months PFS	$268,750	Not mentioned	$88,741 per QALY	$100,000	CE
Muston et al.	Olaparib vs. surveillance (BRCAmut)	2.93 QALYs	$152,545	Not mentioned	$51,986 per QALY; $42,032 per LY	$100,000	CE
Penn et al.	Olaparib vs. observation (BRCAmut)	2.23 PF-LYS	$415,798	No discounting	$186,777 per PF-LYS	$100,000	Not CE
	Olaparib–bevacizumab vs. observation (BRCAmut)	1.48 PF-LYS	$542,708	No discounting	$366,199 per PF-LYS	$100,000	Not CE
	Bevacizumab vs. observation (BRCAmut)	0.26 PF-LYS	$130,541	No discounting	$508,434 per PF-LYS	$100,000	Not CE
	Niraparib vs. observation (BRCAmut)	0.46 PF-LYS	$489,176	No discounting	$1,069,627 per PF-LYS	$100,000	Not CE
	Olaparib–bevacizumab vs. observation (HRD-positive, non-BRCAmut)	0.86 PF-LYS	$542,708	No discounting	$629,347 per PF-LYS	$100,000	Not CE
	Bevacizumab vs. observation (HRD-positive, non-BRCAmut)	0.18 PF-LYS	$130,541	No discounting	$717,255 per PF-LYS	$100,000	Not CE
	Niraparib vs. observation (HRD-positive, non-BRCAmut)	0.46 PF-LYS	$489,176	No discounting	$1,072,754 per PF-LYS	$100,000	Not CE
	Olaparib–bevacizumab vs. observation (HRD-positive)	0.25 PF-LYS	$542,708	No discounting	$2,153,600 per PF-LYS	$100,000	Not CE
	Bevacizumab vs. observation (HRD-positive)	0.23 PF-LYS	$130,541	No discounting	$557,865 per PF-LYS	$100,000	Not CE
	Niraparib vs. observation (HRD-positive)	0.05 PF-LYS	$489,176	No discounting	$10,870,576 per PF-LYS	$100,000	Not CE
Tan et al.	Olaparib vs. routine surveillance (BRCAmut)	2.85 QALYs	$41,184	3% yearly	$14,470 per QALY	$36,496	CE
“Treat-all” vs. biomarker-directed strategy							
Gonzalez et al.	Niraparib-for-all vs. biomarker-directed niraparib	Not mentioned	$68,081	3% yearly	$593,250 per QA-PFY	$150,000	Not CE
	Olaparib/bevacizumab-for-all vs. biomarker-directed olaparib/bevacizumab	Not mentioned	$105,836	3% yearly	$3,347,915 per QA-PFY	$150,000	Not CE

All monetary values were converted to U.S. dollars, using specified exchange rates in publication or average exchange rates in the corresponding year of publication. CE = cost-effective, Not CE = not cost-effective. Abbreviation: BRCAmut = BRCA mutation; HRD = homologous recombination deficiency; IncreCost = incremental cost, IncreEff = incremental effectiveness; LYS = life-year saved; PARPi = poly(ADP-ribose) polymerase inhibitors; PF = progression-free; PFS = progression-free survival; QALY = quality-adjusted life year; WTP = willingness-to-pay.

**TABLE 4:** Details of economic evaluation outcomes of studies in other cancers.

Study	Comparison arms	IncreEff	IncreCost	Discount rate	ICER	WTP threshold	Conclusion
Germline BRCA-mutated, HER2-negative locally advanced or metastatic breast cancer							
Saito et al.	Olaparib for BRCAmut patients after BRCA testing vs. standard chemotherapy alone	0.037 QALY	$4,787	2% yearly	$131,047 per QALY	One- to three-times the GDP of Japan	Not CE
Lima et al.	Talazoparib vs. capecitabine	0.26 QALY	$65,766	Not mentioned	$287,822 per QALY	$23,945	Not CE
	Talazoparib vs. eribulin	0.26 QALY	$67,639	Not mentioned	$296,020 per QALY	$23,945	Not CE
Germline BRCA-mutated metastatic pancreatic cancer							
Wu et al.	Olaparib vs. placebo	0.483 QALY	$128,266	8% yearly	$191,596 per PFS-QALY	$200,000	CE
Zhan et al.	Olaparib vs. placebo	0.69 QALY	$23,544	3% yearly	$34,122 per QALY	$28,256	Not CE
Germline BRCA-mutated, HER2-negative locally advanced or metastatic breast cancer							
Su et al.	Olaparib vs. standard care (at least one of the BRCA1, BRCA2 and ATM gene alterations)	0.063 QALY	$7,382	3% yearly	$116,903 per QALY	$150,000 per QALY	CE
	Olaparib vs. standard care (at least one of the BRCA1, BRCA2, ATM, BRIP1, BARD1, CDK12, CHEK1, CHEK2, FANCL, PALB2, PPP2R2A, RAD51B, RAD51C, RAD51D, and RAD54L gene alterations)	0.068 QALY	$-6,950	3% yearly	Dominated	$150,000 per QALY	CE (olaparib-dominant)

All monetary values were converted to U.S. dollars, using specified exchange rates in publication or average exchange rates in the corresponding year of publication. CE = cost-effective; Not CE = not cost-effective. Abbreviation: BRCAmut = BRCA mutation; HRD = homologous recombination deficiency; IncreCost = incremental cost; IncreEff = incremental effectiveness; LY = life-year; LYS = life-year saved; MLG = month of life gained; PARPi = poly(ADP-ribose) polymerase inhibitors; QALY = quality-adjusted life year; WTP = willingness-to-pay.

### 3.3 Cost and Clinical Benefits of PARP Inhibitors

Overall, the maintenance strategies with PARP inhibitors generated additional 0.07–4.41 QALYs compared with observation. As active treatment, PARP inhibitors gave 0.03–0.67 QALY compared with the standard care. Clinical benefits varied across types and lines of PARP inhibitors, comparison arms, genetic characteristics, time horizon, and simulation methods of survival data. Costs varied greatly between health systems and country contexts.

#### 3.3.1 Treating Recurrent Advanced Ovarian Cancer

PARP inhibitors were widely studied as maintenance treatment for patients with recurrent advanced ovarian cancer who were responsive to platinum-based chemotherapy. Eight studies compared PARP inhibitors against observation, with six of these concluding that they were not cost-effective. Specifically, Smith et al. (2015) determined that olaparib costs $600,552 per PF-YLS for BRCA wild-type patients, with improved ICER at $258,864 per PF-YLS when restricted to gBRCAmut patients, but this was still beyond the $50,000 WTP threshold. Zhong et al. (2018) later expanded the comparison to both olaparib and niraparib and identified their ICERs at $287,000 and $235,000 per PFS-LYS, which dropped only slightly to $197,000 and $226,000 per PFS-LY when restricted to gBRCAmut patients. Consistent findings were noted in the Institute for Clinical and Economic Review Report, two more studies in the United States (Secord et al., 2013; Institute for Clinical and Economic Review 2017; Dottino et al., 2019), and one study in the Singaporean context (Cheng et al., 2021). The Guy group in the United States was one of the two that concluded PARP inhibitors were cost-effective in comparison to observation, with niraparib in both gBRCAmut and wild-type patients giving ICERs at $68,287 and $108,287 per QALY, albeit a questionably high WTP threshold at $150,000 (Guy et al., 2019). Another similar conclusion was drawn in Taiwan by Leung et al., which later expanded the comparison to both niraparib and olaparib. Regardless of genetic features, the ICERs accounted for 69–90% of the WTP threshold. When restricted to gBRCAmut patients only, the ICERs even dropped to between 28 and 43% of the threshold (Leung et al., 2021).

In contrast to maintenance therapies, two studies evaluated the active role of PARP inhibitors in later lines in comparison with chemotherapies in the recurrence setting, with inconsistent results. In BRCAmut patients, the Institute for Clinical and Economic Review Report deemed olaparib cost-effective versus PLD + C (pegylated liposomal doxorubicin + carboplatin) at $146,210 per QALY, in contrast to rucaparib, which was not cost-effective at $294,593 per QALY against PLD + C (Institute for Clinical and Economic Review 2017). However, the genome-guided approach with next-generation sequencing (NGS) to test all concurrent targetable mutations was not cost-effective, although it was in the context of platinum-resistant cases (Wallbillich et al., 2016).

#### 3.3.2 Treating Newly Diagnosed Advanced Ovarian Cancer

Since 2018, PARP inhibitors have been approved for earlier use as upfront maintenance rather than waiting for relapse occurrence following successful response to first-line chemotherapy, based on genetic characteristics. Six studies investigated their value as upfront maintenance. Five studies compared PARP inhibitors against observation with four concluding them to be cost-effective. In particular, Tan et al. (2021)compared first-line olaparib maintenance with routine surveillance in the Singaporean context and demonstrated $14,470 per QALY, an ICER far below their $36,496 WTP threshold. Olaparib was similarly found to be cost-effective in Italy and the United States, with ICERs accounting for 52–69% of their WTP thresholds (Armeni et al., 2020; Muston et al., 2020). Apart from olaparib, Barrington et al. (2020) comprehensively modeled five scenarios when offering niraparib to all patients, HRD-positive-only patients, BRCAmut-only patients, HRD-positive non-BRCAmut patients, and non–HRD-positive patients; all ICERs ranged from $50,914 to $88,741 per QALY, which remained lower than the $100,000 threshold. Without applying cost and benefit discounting, Penn et al. was the only group that showed negative findings for first-line maintenance with and without adding an antiangiogenic agent. Among the ten strategies composed of olaparib-only, niraparib-only, bevacizumab-only, and olaparib and bevacizumab with and without stratification by genetic characteristics, when compared against observation, the ICERs stayed high in the range from $366,100 to $10,870,576 per PF-LYS (Penn et al., 2020).

#### 3.3.3 Treating Metastatic Breast, Pancreatic, and Prostate Cancers

PARP inhibitors were approved for metastatic breast cancer with HER2-negative gBRCAmut patients who failed chemotherapy or for HR-positive gBRCAmut patients who failed or were ineligible for endocrine therapies. Two studies investigated the cost-effectiveness of PARP inhibitor versus standard chemotherapies in these patients, and both deemed the former not cost-effective. Saito’s group in Japan compared the strategy of olaparib monotherapy after positive BRCA mutation profiling to the use of capecitabine, eribulin, or vinorelbine without testing and discovered that the former costs $131,047 per QALY, which was hardly cost-effective at the WTP threshold of $89,286 (Saito et al., 2019). Regarding talazoparib, consistent findings were presented in a Spanish study by Lima and others, who obtained ICERs slightly above $280,000 per QALY in two scenarios compared with capecitabine or eribulin, which were ten-fold higher than the threshold of $23,945 (Olry de Labry Lima et al., 2021).

Olaparib, as maintenance after first-line platinum-based chemotherapy for g*BRCA*mut metastatic pancreatic cancer, a recent indication approved in 2019, was studied by two groups for its cost-effectiveness versus placebo, but the conclusions were mixed. In particular, Wu and Shi (2020) in the United States found that olaparib was cost-effective with an ICER at $191,596 per PFS-QALY, which was below the $200,000 threshold but not the case when modeling was based on overall survival data ($265,290 per QALY). In China, Zhan et al. (2020) identified an ICER at $34,122 per QALY which did not support cost-effectiveness at the WTP threshold of $28,255, although the ICER drastically dropped to $14,563 per QALY when the drug cost was calculated based on the donation plan for ovarian cancer.

Only one study evaluated the cost-effectiveness in metastatic castration-resistant prostate cancer, owing to the recent approval in 2020. In the two scenarios modeled by Su et al. in the United States, compared with the standard care, olaparib was cost-effective when used among patients with at least one gene alteration in BRCA1, BRCA2, and ATM, with an ICER yielded at $116,903 per QALY. In the case of expanding the treatment group to patients who had alterations in any of all 15 pre-specified genes (BRCA1/2, ATM, BRIP1, BARD1, CDK12, CHEK1/2, FANCL, PALB2, PPP2R2A, RAD51B/C/D, and RAD54L) after NGS testing, olaparib turned out to be a cost-effective option (Su et al., 2020).

### 3.4 Worthiness of the Biomarker-Directed Treatment Strategy

In the studies that separated their analyses based on genetic characteristics, the ICERs using PARP inhibitors among only BRCAmut and/or HRD-positive patients were always lower than those among all patients, implying that the biomarker-directed treatment strategy was potentially cost-effective. Four studies directly compared the biomarker-directed strategy against “global PARP inhibitors,” an approach that offers the drug to all patients, regardless of their genetic characteristics. In recurrent advanced ovarian cancer, Secord et al. (2013)concluded that “global olaparib” offered the greatest efficacy but was the costliest. In the United States and Singapore, “global olaparib” was associated with an incremental cost of $234,128 or $105,300 per progression-free life year compared with the BRCA1/2 testing stratification strategy (Secord et al., 2013; Cheng et al., 2021). However, compared with observation only, BRCA1/2 testing-directed treatment was still not cost-effective. The Dottino group demonstrated similar findings for niraparib, except that they also evaluated the addition of HRD testing alongside BRCA testing before treatment (Dottino et al., 2019). Consistently in newly diagnosed advanced ovarian cancer, Gonzalez et al. (2020) concluded that when compared with biomarker-directed treatment, adopting “global olaparib or niraparib” yielded ICERs as high as $3,347,915 per QA-PFY.

### 3.5 Key Cost-Effectiveness Determinants

All the articles performed sensitivity analyses to assess the factors which potentially impacted the cost-effectiveness of PARP inhibitors. Out of 20 studies, 17 highlighted that the drug price was a significant driver of the ICER (Secord et al., 2013; Smith et al., 2015; Wallbillich et al., 2016; Institute for Clinical and Economic Review, 2017; Zhong et al., 2018; Dottino et al., 2019; Armeni et al., 2020; Barrington et al., 2020; Gonzalez et al., 2020; Muston et al., 2020; Penn et al., 2020; Su et al., 2020; Wu and Shi, 2020; Zhan et al., 2020; Cheng et al., 2021; Leung et al., 2021; Olry de Labry Lima et al., 2021). In the United States system, to be cost-effective in treating recurrent ovarian cancer among BRCAmut patients, major cost reduction to $3,000–6,400 per cycle was warranted for olaparib, niraparib, and rucaparib, which was up to 76% reduction at the WTP of $100,000 (Secord et al., 2013; Smith et al., 2015; Wallbillich et al., 2016; Dottino et al., 2019). For newly diagnosed ovarian and metastatic pancreatic cancers, 47 and 50% reduction in olaparib cost for BRCAmut patients was highlighted, respectively (Penn et al., 2020; Wu and Shi, 2020). In the Spanish context, for metastatic breast cancer, an even more drastic price cut for talazoparib to $906 per cycle (85% reduction) was warranted to reach the $23,945 threshold (Olry de Labry Lima et al., 2021). ICERs were less sensitive to the costs of chemotherapies, hospital care, general adverse event management, and molecular testing. As for clinical estimates, models were more sensitive to the hazard ratios of PFS or the ratios used to project overall survival, time-receiving maintenance treatment, and utility values at progressive disease state (Smith et al., 2015; Zhong et al., 2018; Guy et al., 2019; Saito et al., 2019; Armeni et al., 2020; Barrington et al., 2020; Su et al., 2020; Wu and Shi 2020; Leung et al., 2021).

## 4 Discussion

PARP inhibitors marked a breakthrough in the burgeoning wave of precision oncology as they provide substantial progression-free survival benefit in a broad range of patients with actionable targets. The unbridled high costs may nonetheless hinder their presence in clinical routines; health economic studies are therefore warranted to assess priorities. This systematic review depicts several findings. First, the cost-effectiveness of PARP inhibitors varied with cancer types and lines of treatment. In most cases, they were not cost-effective as maintenance treatment for recurrent advanced ovarian cancer was compared with observation, but a stronger potential was attained when moved earlier to upfront maintenance in newly diagnosed advanced ovarian cancer. Limited evidence showed that PARP inhibitors were not cost-effective in metastatic breast cancer. The conclusions were mixed for metastatic pancreatic cancer, whilst olaparib in metastatic prostate cancer seemed to be cost-effective. Next, stratification by tumor genetic characteristics displayed an effect on ICERs, given the plummeting ICER values after confining treatment to BRCAmut- and/or HRD-only patients. Finally, drug cost was consistently highlighted in all models as a strong cost-effectiveness determinant, followed by the hazard ratio of PFS in some models. However, costs of comparator treatments, hospice care, general adverse event management, and molecular tests made minimal impact on all models.

This review serves to inform payers of the overall cost-effectiveness pattern of PARP inhibitors and key areas to intervene for resource prioritization. Although all included studies utilized registrational randomized data, the overall conclusions in the analyses are logical. In platinum-sensitive BRCAmut ovarian cancer, olaparib maintenance offered significant PFS improvement with 19.1 versus 5.5 months of placebo upon first recurrence in the landmark trial, but when the drug was moved to upfront maintenance in newly diagnosed advanced ovarian cancer, the estimated median PFS difference was numerically extended to 36 months (Pujade-Lauraine et al., 2017; Moore et al., 2018). Despite the higher cost, cost-effectiveness was reflected by the greater survival difference for earlier use along the patient’s journey. This finding echoes the recent guidance from the National Institute for Health and Care Excellence in 2020 and 2021 that olaparib alone or plus bevacizumab should be recommended for first-line maintenance in Cancer Drugs Fund with managed access agreement (National Institute for Health and Care Excellence 2019; National Institute for Health and Care Excellence 2021), heralding the importance of maximizing the value of the drug by re-adjusting the treatment position of PARP inhibitors and identifying BRCA mutation and HRD early at the time of diagnosis. In all relevant articles, biomarker-directed treatment was always more cost-effective than treating all patients with PARP inhibitors, regardless of genetic features. Comprehensive genome profiling was particularly valuable in metastatic prostate cancer as the targeted use of olaparib among patients with alterations in any of the 15 pre-specified genes yielded a cost-effective option, which was concluded as a more appropriate strategy than testing for only three gene alterations, owing to a lower number needed to screen for identifying eligible patients. When a funding decision has to be made, it is important to prioritize targeted use based on genetic stratification and to select the composition of test panels prudently to maximize the value of PARP inhibitors.

Undeniably, the poor cost-effectiveness of PARP inhibitors in recurrent ovarian cancer and metastatic breast cancer remains an issue. The standard comparator in platinum-sensitive ovarian cases was wait-and-watch, which explains the tremendous incremental cost, following the introduction of an extra treatment. In the case of metastatic breast cancer, however, it was more attributed by the relatively minuscule incremental QALY as olaparib and talazoparib were compared against an effective treatment. Another contributor for both was the steep drug price, a strong determining factor identified in 85% of the studies. Taking the United States system as an example, the per month wholesale acquisition costs of PARP inhibitors for ovarian cancer ranged from $13,679 to 18,070 between 2017 and 2018 (Institute for Clinical and Economic Review 2017; Gonzalez et al., 2020). In this review, PARP inhibitors could face a radical price cut to as low as $3,000 in order to fulfill the common WTP thresholds at $100,000, but the requirement seems unrealistic since other novel targeted therapies were commonly marketed at $5,000–10,000 per month or higher (Kaplan, 2017). Price negotiation could be an alternative measure as Zhan et al. (2020) found that olaparib turned out to be cost-effective in metastatic pancreatic cancer despite off-label use if the discounted price approved in ovarian cancer could similarly be applied to pancreatic cancer (Zhan et al., 2020).

There is a disproportionate distribution of economic evidence across different indications, countries, and health systems. The majority of studies were in the United States, and the remaining studies mostly originated from other developed countries, which signify an unmet need in developing countries where cost-effectiveness or even treatment access is questionable. Next, compared with advanced ovarian cancer, fewer studies evaluated the use of PARP inhibitors in metastatic breast, pancreatic, and prostate cancers, given that the latter indications were only approved recently. An assessment of cost-effectiveness consistency across systems was therefore less possible in these indications. Finally, owing to the lack of mature overall survival data, most studies either projected the overall survival impact based on the available PFS data or relied heavily on PF-QALY for interpretation. However, previous literature studies showed that a positive PFS correctly predicted a positive overall survival only 71% of the time (Lakdawalla et al., 2015). Since overall survival depicts the actual length of time until death, it is of greater clinical importance and accounts for any diminished effect in subsequent therapies after PARP inhibitor treatment. Therefore, further verification with mature data from trial or real-world evidence is highly encouraged as this will be critical for payers to confirm how PFS could be translated into overall survival benefit.

## 5 Conclusion

PARP inhibitors were not cost-effective as maintenance treatment for recurrent ovarian cancer but could be cost-effective if used for newly diagnosed patients. PARP inhibitor use should be prioritized for upfront maintenance and for patients with BRCA mutation or BRCAness at recurrence. Economic evidence in metastatic breast, pancreatic, and prostate cancers was less and with mixed conclusions. Drug cost is the most important determinant for cost-effectiveness. Additional economic evaluations across the globe with mature overall survival data and novel indications are anticipated.

## Data Availability

The original contributions presented in the study are included in the article/Supplementary Material; further inquiries can be directed to the corresponding author.
